# *In Situ* Transplantation of Alginate Bioencapsulated Adipose Tissues Derived Stem Cells (ADSCs) via Hepatic Injection in a Mouse Model

**DOI:** 10.1371/journal.pone.0138184

**Published:** 2015-09-15

**Authors:** Mong-Jen Chen, Yuanqing Lu, Nicholas E. Simpson, Mark J. Beveridge, Ahmed S. Elshikha, Mohammad Ahsanul Akbar, Hsin-Yin Tsai, Stephanie Hinske, Junling Qin, Christian R. Grunwitz, Tina Chen, Mark L. Brantly, Sihong Song

**Affiliations:** 1 Department of Pharmaceutics, College of Pharmacy, University of Florida, Gainesville, Florida, United States of America; 2 Department of Medicine, University of Florida, Gainesville, Florida, United States of America; Rutgers University -New Jersey Medical School, UNITED STATES

## Abstract

**Objective:**

Adipose tissue derived stem cells (ADSCs) transplantation has recently gained widespread enthusiasm, particularly in the perspective to use them as potential alternative cell sources for hepatocytes in cell based therapy, mainly because of their capability of hepatogenic differentiation *in vitro* and *in vivo*. But some challenges remain to be addressed, including whether ADSCs can be provided effectively to the target organ and whether subsequent proliferation of transplanted cells can be achieved. To date, intrasplenic injection is the conventional method to deliver ADSCs into the liver; however, a number of donor cells retained in the spleen has been reported. In this study, our objective is to evaluate a novel route to transplant ADSCs specifically to the liver. We aimed to test the feasibility of *in situ* transplantation of ADSCs by injecting bioencapsulated ADSCs into the liver in mouse model.

**Methods:**

The ADSCs isolated from human alpha 1 antitrypsin (M-hAAT) transgenic mice were used to allow delivered ADSCs be readily identified in the liver of recipient mice, and alginate was selected as a cell carrier. We first evaluated whether alginate microspheres are implantable into the liver tissue by injection and whether ADSCs could migrate from alginate microspheres (study one). Once proven, we then examined the *in vivo* fate of ADSCs loaded microspheres in the liver. Specifically, we evaluated whether transplanted, undifferentiated ASDCs could be induced by the local microenvironment toward hepatogenic differentiation and the distribution of surviving ADSCs in major tissue organs (study two).

**Results:**

Our results indicated ADSCs loaded alginate microspheres were implantable into the liver. Both degraded and residual alginate microspheres were observed in the liver up to three weeks. The viable ADSCs were detectable surrounding degraded and residual alginate microspheres in the liver and other major organs such as bone marrow and the lungs. Importantly, transplanted ADSCs underwent hepatogenic differentiation to become cells expressing albumin in the liver. These findings improve our understanding of the interplay between ADSCs (donor cells), alginate (biomaterial), and local microenvironment in a hepatectomized mouse model, and might improve the strategy of *in situ* transplantation of ADSCs in treating liver diseases.

## Introduction

Management of patients with acute and chronic hepatic failure is complex and expensive. Many such end stage liver diseases can only be treatable today by liver transplantation. Unfortunately, the use of whole liver transplantation to treat these disorders is limited by a severe shortage of donors and by the risks to the recipient associated with major surgery [1]. Recently, a number of studies on rodent models indicated that transplants consisting of isolated hepatocytes can correct various metabolic deficiencies of liver and reverse liver hepatic failure [2–4]. However, its applicability remains limited by a number of issues, such as the shortage of hepatocytes, high cost, and relatively poor initial and long-term hepatocyte engraftment in the recipient [1].

The adipose tissue-derived stem cells (ADSCs) are mesenchymal stem cells which have been shown to have hepatogenic capability *in vitro* and *in vivo* [5–7] and actions of repair to liver damages [8, 9]. The mechanism of actions was not clearly elucidated but may include their ability to differentiate into hepatocyte-like cells, to reduce inflammation, and to enhance tissue repair at the site of injury. These unique characteristics make them a suitable alternative cell source for hepatocytes in a cell based therapy [7, 10]. To date, splenic injection is the conventional method to transplant ADSCs into the liver. The donor cells migrated toward sinusoids because splenic blood drains into the portal vein [11]. However, a number of donor ADSCs was reported to remain in the spleen few weeks after transplantation [12]. This indicated a loss of donor cells and could possibly lead to unwanted side effects at non-target organs. To maximize the number of donor cells which could be locally delivered to the liver, we developed a strategy of *in situ* transplantation, in which donor ADSCs are bioencapsulated into a biomaterial and then transplanted directly into the liver tissue by simple injection.

Alginate was selected as the cell carrier in this study to reduce the possible cell loss due to excessive shear stress during the syringe injection and to maximize the amount of delivered ADSCs. Alginates are natural, linear unbranched polysaccharides with unique properties, including gentle gelation behavior, biodegradability, biocompatibility, and ease of cell encapsulation. A number of studies have demonstrated ADSCs can be readily cultured, encapsulated, and injected in alginate microspheres [13]. Application of alginate bioencapsulated ADSCs have been used in in the repair of myocardial infarction in the rat model [14] and improving bone regeneration [15]. Recently, human bone marrow-derived mesenchymal stem cells (BM-MSC) have also been used by the similar technology to show MSC-derived soluble molecules decreased experimental liver fibrosis in mice [16]. However, the transplantation of alginate bioencapsulated ADSCs into the liver has never been assessed.

The purpose of this study is to test the feasibility of *in situ* ADSCs transplantation by injecting bioencapsulated ADSCs into the liver in a hepatectomized mouse model. Our aim was to determine whether alginate microspheres could be used to locally deliver ADSCs to the liver via injection. Once proven, we then examined the *in vivo* fate of ADSCs loaded microspheres in the liver, evaluated the hepatogenic differentiation of undifferentiated ADSCs in local microenvironment, and examined the distribution of survived ADSCs in major tissue organs.

## Materials and Methods

### Cell isolation and culture

The isolation of ADSCs was performed as previous described [17]. In brief, the stromal vascular fraction (SVF) was primarily isolated from white adipose tissue of human M-hAAT transgenic mice (4 weeks old). Adipose tissue was first enzymatically digested with 0.075% type I collagenase (Sigma, St. Louis, MO) in PBS for 1hr at 37°C with gentle agitation. The collagenase was then inactivated with an equal volume of DMEM supplemented with 10% fetal bovine serum (FBS) and the suspension was centrifuged at 1000g for 5 min at room temperature. The resulting cell pellet was resuspended in 160 mM NH4Cl, incubated at room temperature for 2 min to eliminate contaminating red blood cells and filtered through a 100μm nylon mesh strainer to remove debris. The resulting ADSCs containing cell pellet was collected by centrifugation as described above, resuspended in a DMEM/10% FBS medium, and plated on plastic tissue culture dishes. Adherent cells were cultured in DMEM/10% FBS medium supplemented with 5ng/mL bFGF under hypoxic condition (5%O2 and 5%CO2) for expansion; hypoxic condition and bFGF have been shown to increase ADSCs proliferation [18, 19]. In addition, ADSCs isolated from a mutant (Z-hAAT) transgenic mice, in which Z-hAAT is controlled by the liver specific promoter, and mouse embryonic fibroblast (MEF) which were primary isolated from C57BL/6J mice embryo were used as negative control for downstream ELISA experiments; MEF is a kind gift from Dr. Terada at the University of Florida.

### Characterization of transgenic ADSCs in vitro

FACS analysis: Fluorescence activated cell sorting (FACS) analysis of isolated mouse ADSCs (passages 3) was performed using a BD LSRII analyzer (BD bioscience, New Jersey) at the University of Florida FACS facility core. Antibodies used in FACS analysis were FITC-conjugated anti CD90, CD105, CD31, and CD45. All were purchased from eBiosciences (San Diego, CA, USA).

ELISA: To ensure cultured transgenic ADSCs secreted hAAT, cell culture medium harvested from ADSCs (both M-hAAT- and Z-hAAT transgenic mice), and cell culture medium harvested from mouse embryonic fibroblast (MEF) were collected at passage 3 and analyzed by anti-hAAT ELISA [12]. MEF is used as a nonspecific negative control to show the background reading of ELISA. The ADSCs from Z-hAAT transgenic mice is served as a negative control of ADSCs from M-hAAT transgenic mice since hAAT gene is not active in Z-hAAT transgenic mice. Mouse albumin ELISA was performed according to manufacturer’s instruction (Kamiya Biomedical Company, Seattle, WA, USA) to exclude albumin expression of ADSCs isolated from M-hAAT transgenic mouse.

### Production of calcium alginate microspheres in vitro

The low viscosity, high mannuronic acid (LVM) alginate solution (2% wt/vol) was made by dissolving alginate LVM (NovaMatrix, Philadelphia, PA) in PBS without calcium overnight and storing at 4°C before use. Next, the 2% wt/vol LVM alginate solution was loaded into a syringe pump and connected to an electrostatic bead generator (Nisco Engineering AG, Zurich, Switzerland). Alginate microspheres were produced with a flow rate of 0.35 ml/min, voltage of 6.0 kV, 350nm gauge nozzle, and CaCl_2_ gelling bath solution with a concentration of 102 mM. The mean diameter of the prepared alginate microsphere was determined as 250 μm. After formation, the microspheres were washed by PBS with calcium twice to remove excess CaCl_2_ and harden the microspheres, and then washed by cell culture medium (DMEM supplemented with 10% FBS). After washing, the cell culture medium was removed and the alginate microspheres were stored in fresh cell culture medium at 37°C in the incubator on a shaker for future use.

### Preparation of alginate bioencapsulated ADSCs in vitro

The preparation method for alginate bioencapsulated ADSCs was similar to conventional alginate microsphere preparation as mentioned above. Briefly, the ADSCs cell suspension was added into the alginate solution to have a final cell density of 5 x 10^6^ cells per ml. The cell-alginate solution was well mixed and then transferred to the syringe. The microsphere production procedure was performed quickly under sterile conditions to ensure the cell viability and to prevent contamination. The alginate microspheres containing ADSCs were washed twice by PBS with calcium to harden the microspheres, and then washed by cell culture medium. To examine ADSCs inside the alginate microspheres, the alginate microspheres were fixed with 2% glutaraldehyde for 2 hours and then embedded in paraffin, and sectioned for standard H&E staining. The prepared bioencapsulated ADSCs were used for cell transplantation within 24 hours after microencapsulation, and each animal in experimental groups was given 0.2 ml alginate microspheres suspension, which is equivalent to one million cells per injection.

### Determine glucose concentration in the cell culture medium

One mL alginate microspheres (5 million cell per ml) was added into 6 well cell culture plate. The cell culture medium were collected and designated as t0 (fresh medium) and t1 (after two days culture) every two days. The glucose concentration in the cell culture medium was determined by VITROS DT60 II (Ortho-Clinical Diagnostics, Rochester, NY) according to manufacturer’s manual. The glucose consumption (per 5 million cells in two days) was determined by the difference of glucose concentration between two selected time points (t1- t0). Each sample was analyzed in triplicate. The decrease of glucose concentration in cell culture medium serves as an indicator of cell metabolic activity.

### Ethics Statement

All the animal procedures including isolation of ADSCs and cell transplantation were approved by the University of Florida Institutional Animal Care and Use Committee (IACUC). The isolation of ADSCs was performed as previously described [12].

### Animal studies

A series of animal experiments were conducted in the present study. For each animal 2/3 partial hepatectomy (PH) was performed [20] to create liver damage to trigger liver regeneration microenvironment in attempt to induce hepatogenic differentiation of transplanted ADSCs. We have tested different sizes of needles to minimize the possible liver tissue damage due to injection and 22 gauge needles were selected to be used in delivering alginate microspheres suspensions. In study one, we aimed to evaluate whether alginate microspheres are implantable into the liver tissue by injection. Six hepatectomized male C57BL/6J mice were randomized and divided into 3 groups (n = 2 in each group): Group P1 (single dose of 1 million bioencapsulated ADSCs via hepatic injection), Group P2 (single dose of 1 million bioencapsulated ADSC via hepatic injection), Group P3 (single injection of PBS). All were injected with a volume of 0.2 ml. Animals in Group P1 were sacrificed 2 hours after cell transplantation, while animals in Group P2 and P3 were sacrificed 2 weeks after cell transplantation.

Subsequently, a second animal study (study two) was performed to evaluate the hepatogenic differentiation of ADCSs in the liver and distribution of survived ADSCs in other major tissue organs. In brief, 12 hepatectomized male C57BL/6J mice were randomized and divided into 3 groups (n = 4 in each treatment group): Group 1 (single dose of 1million bioencapsulated ADSCs via hepatic injection), Group 2 (single dose of 1million free ADSCs via intrasplenic injection), and Group 3 (single injection of empty alginate microspheres via hepatic injection). Animals were sacrificed 3 weeks after transplantation; major organ tissues, including pancreas, brain, kidney, muscle, testes, intestine, spleen, lung, and bone marrow, and muscle were isolated and collected for histological examination (immunostaining against hAAT).

### Detection of anti-hAAT antibody

To assess if hAAT would induce generation of anti-hAAT antibody in recipient mice, the blood level of anti-hAAT antibody was determined 3 weeks after transplantation by using anti-hAAT antibody ELISA [12].

### Immunohistochemistry

The organ tissues were fixed in 10% neutral buffered formalin (NBF) and then embedded in paraffin. Paraffin-embedded tissue was sliced and stained with hematoxyl and eosin (H&E). Human AAT and mouse albumin were detected by immunostaining as previously described [12]. The anti-hAAT antibody was purchased from Fitzgerald Industries International (North Acton, MA, USA; Cat. No. 20R-AR009); the anti-albumin antibody was purchased from Abcam (Cambridge, MA, USA; Cat. No. ab19194); the anti-Ki67 antibody was purchased from Dako (Carpinteria, CA, USA, No. M7249).

### Statistical analysis

Statistical analysis was performed with GraphPad Prism versions 5 for Windows. One way ANOVA and Student's t-test were used for comparisons. A significant level of *p* < 0.05 was used to define significant differences. All data are expressed as mean ± s.d.

## Results

### Characterization of adipose-tissue derived stem cells (ADSCs) isolated from human alpha 1 antitrypsin (M-hAAT) transgenic mice

**Fig 1:**
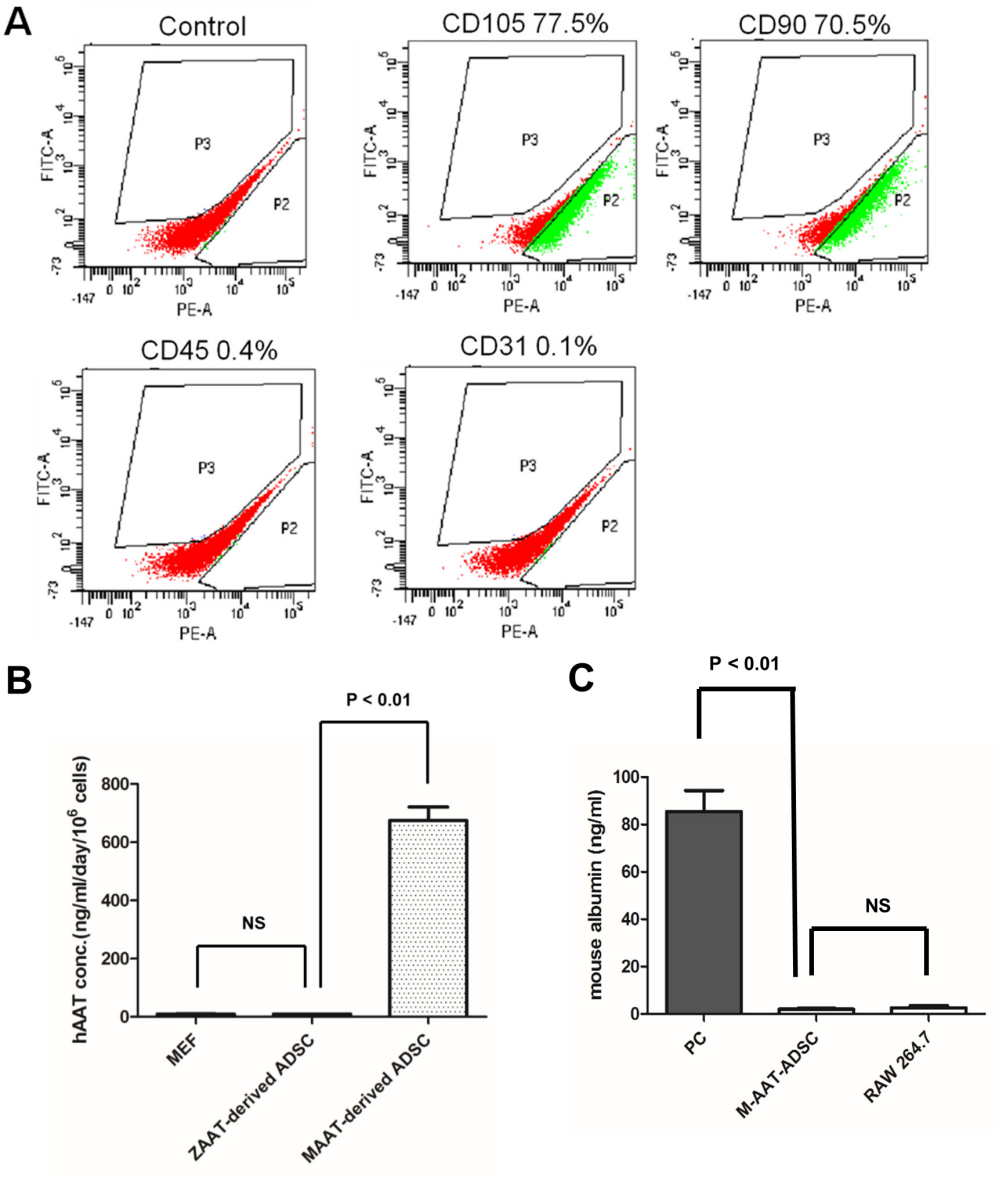
Characterization of adipose tissue derived stem cells (ADSCs) isolated from M-hAAT transgenic mice. (A) Representative pictures of FACS analysis showing isolated mouse ADSC expressed mesenchymal stem cell markers CD90 and CD105, but not hematopoietic lineage markers, such as CD45, or endothelial cell markers, such as CD31; (B) Detection of human AAT protein in cell culture medium by ELISA; (C) Detection of mouse albumin in cell culture medium by ELISA. Mouse albumin was used as a positive control (PC) and cell culture medium from RAW264.7 was used as a negative control.

**Fig 2:**
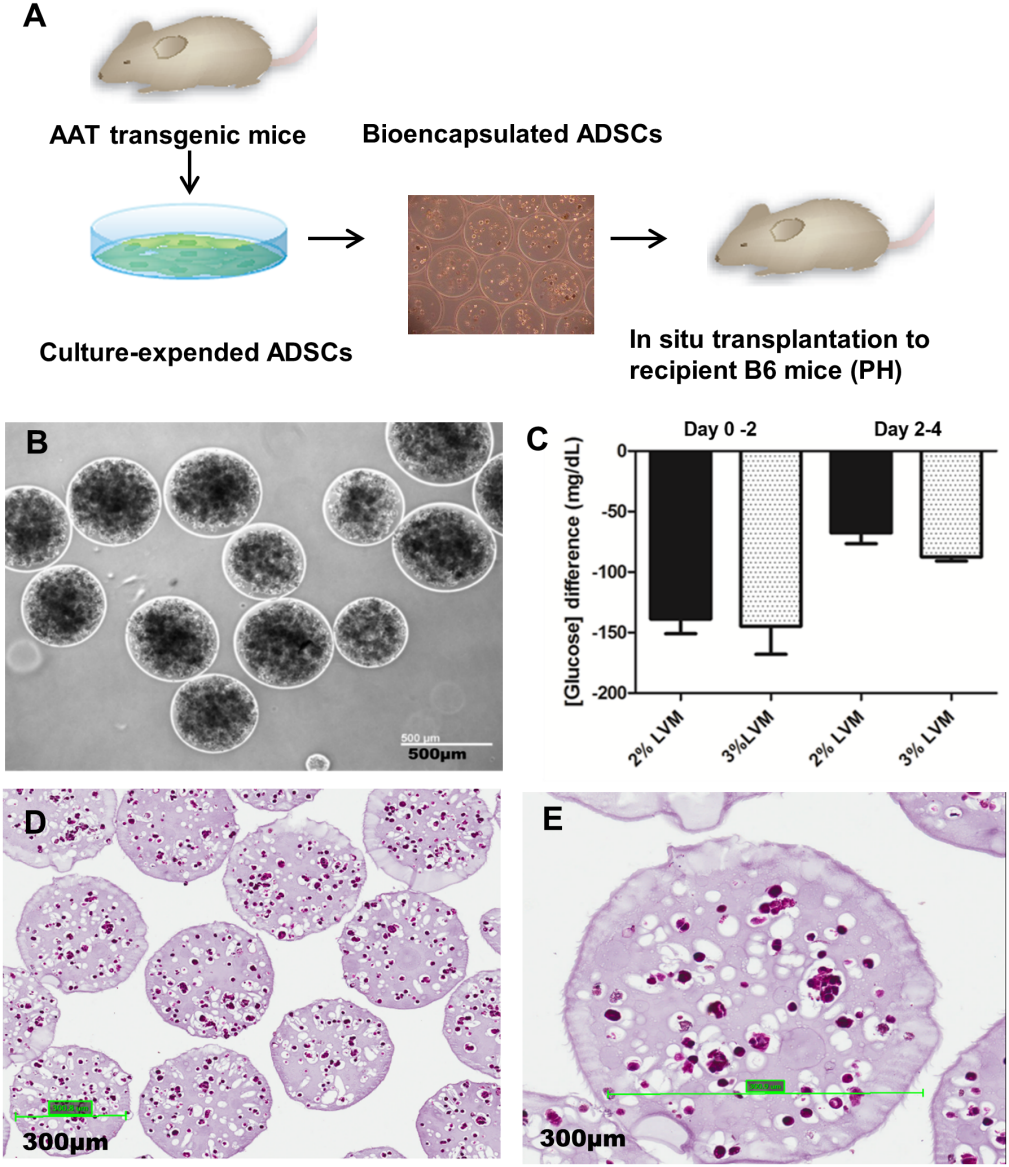
The bioencapsulation of ADSCs *in vitro*. (A) The strategy of *in situ* transplantation to locally deliver ADSCs into the liver tissue; (B) The morphology of alginate microspheres containing ADSCs after production; (C) The metabolic activities of ADSCs after bioencapsulation. The negative values indicated cells uptake glucose from culture medium; (D) The histology of alginate microspheres containing ADSCs (H&E staining); (E) The enlarged picture of picture D.

The ADSCs used in the present study were isolated from white adipose tissue of human alpha 1 antitrypsin (hAAT) transgenic mice. Here we use hAAT as a marker for donor cell identification in the recipient tissues by hAAT specific immunostaining. After completion of isolation processes, the cells from white adipose tissue were seeded on the cell culture plate. The initially adherent cells grew into spindle-shaped cells that developed into visible colonies, and then begin to rapidly proliferate under the hypoxia condition (5% oxygen) in the incubator. We characterized those cells at the third passage by examining lineage specific cell surface markers using fluorescence activated cell sorting (FACS) (Fig 1A). Enriched ADSCs demonstrated distinct mesenchymal surface markers, such as CD90 (70.5%) and CD105 (77.5%), but negative for hematopoietic lineage markers, such as CD45 (0.4%), or endothelial cell marker, such as CD31 (0.1%). To ensure cultured transgenic ADSCs expressed hAAT, we performed ELISA to detect hAAT level in the cell culture medium. We showed transgenic ADSCs secreted hAAT protein into the cell culture medium; no hAAT protein was detectable in the cell culture medium collected from control groups (Fig 1B). Albumin ELISA was performed to confirm ADSCs isolated from hAAT transgenic mouse do not express albumin (Fig 1C). The ADSCs expressing hAAT were then encapsulated into alginate microspheres and injected into the liver tissue (Fig 2A).

### Optimization of production of calcium alginate microspheres containing ADSCs

We performed a series of *in vitro* experiments to optimize the encapsulation processes. In brief, we evaluated different types and concentrations of divalent cations, including calcium (Ca^2+^) and barium (Ba^2+^), for initiating polymerization processes of alginate microsphere, followed by testing different amount of ADSCs which could possibly be packaged into 1 ml LVM solution, including 1, 3, and 5 million cells, in an attempt to reach the highest encapsulation capacity. After processing, we demonstrated bioencapsulated ADSCs were homogenous dispersed in uniform microspheres (Fig 2B). To detect cellular metabolic activities after bioencapsulation, we measured glucose concentration at selected time points (every 48 hours and continued for 4 days) in cell culture medium to determine the glucose consumption level between two different alginate microspheres formulations, including 2% wt/vol and 3% wt/vol LVM. Compared the difference of glucose concentration between Day0-2 (-138.66 ± 21.12 mg/dL) and Day2-4 (-67.66 ± 15.50 mg/dL) in 2% wt/vol LVM, we found about a 50% reduced metabolic activities. Negative values indicated cells uptake glucose from the culture medium. Similarly, in 3% wt/vol LVM, the reduced metabolic activities was observed (Day0-2: -144.66 ± 40.37 mg/dL vs Day2-4: -87.5 ± 4.94 mg/dL). But no statistical significance difference was detected between 2% wt/vol and 3% wt/vol LVM (Fig 2C). This result indicated ADSCs are alive after bioencapsulation but a possible cell loss might happen in a longer *in vitro* cell culture. The histology of bioencapsulated ADSCs showed that ADSCs were evenly distributed within the porous alginate microspheres (Fig 2D and 2E).

Our overall strategy was to allow ADSCs to migrate from the microspheres after transplantation, so soft alginate (LVM) microspheres were used to increase the possibility that donor cells can migrate from alginate microspheres *in vivo*. We thus decided to prepare calcium alginate microspheres by packaging 5 million ADSCs in 1 mL 2% wt/vol LVM. Since our results showed a reduced metabolic activities if bioencapsulated cells cultured *in vitro* for a longer time, to avoid the possible cell loss due to a longer period of *in vitro* cell culture, the prepared alginate microspheres were transplanted to recipient mice within 24 hours and each mouse in alginate microsphere treatment group was given 0.2 ml of alginate microspheres suspension in a concentration of 5 million cells per mL, which is appromaxily equivalent to 1 million ADSCs per mouse.

### Detection of survival of bioencapsulated transgenic ADSCs in the liver

We first evaluated whether alginate microspheres are implantable into the liver tissue via injection and evaluate the integrity of ADSCs loaded microspheres in the liver (study one). In brief, 0.2 ml bioencapsulated ADSCs suspension (equivalent to 1 million cells) was directly injected into the remaining liver lobe after 2/3 PH. Recipient mice were sacrificed at selected time points post-transplantation (2 hours and 2 weeks) and the liver tissues were collected for histology examination.

**Fig 3:**
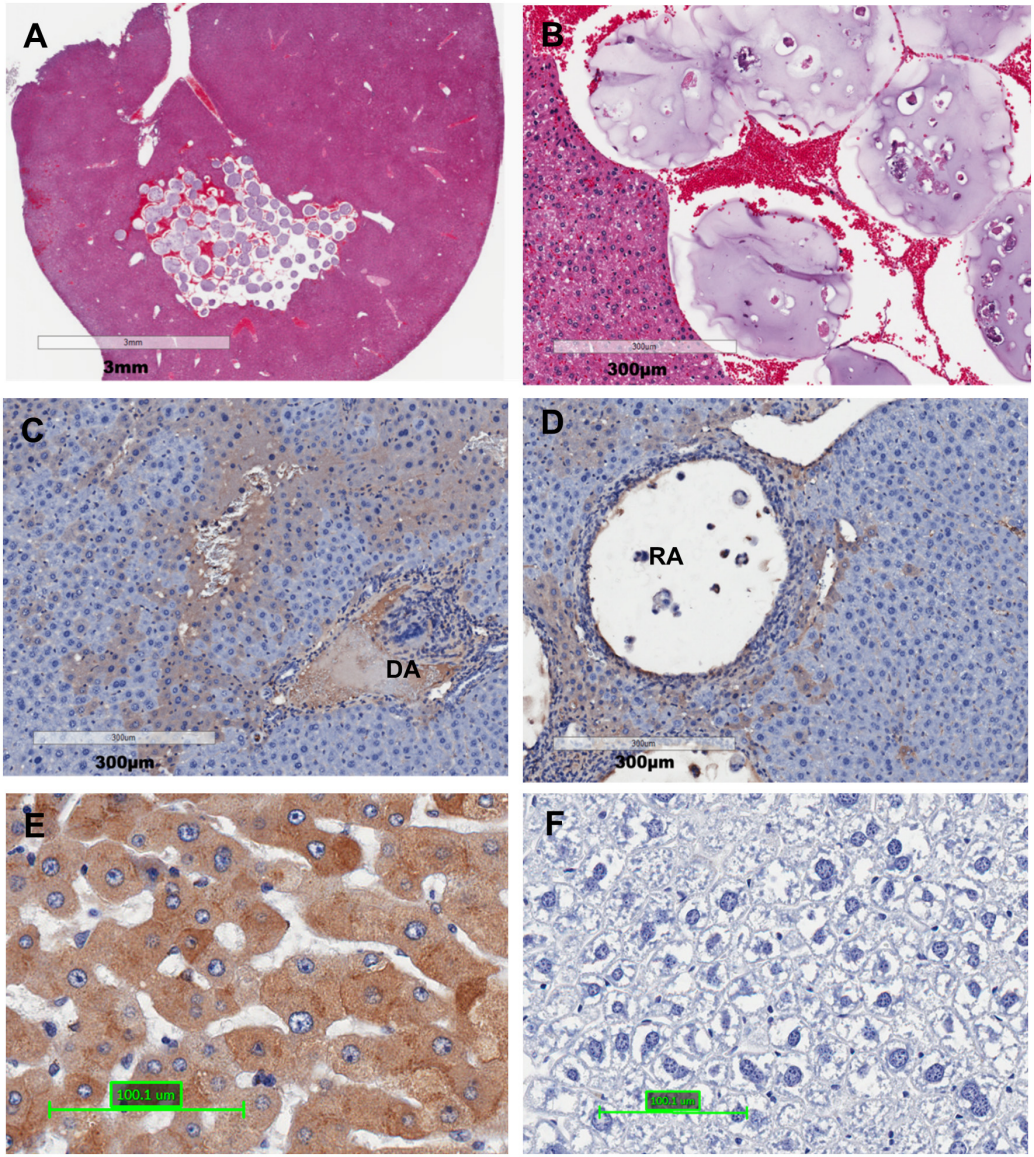
The survival of bioencapsulated ADSCs in the liver 2 weeks after *in situ* transplantation. (A) Detection of intact alginate microspheres 2 hours after transplantation; (B) The enlarged picture of picture A; (C) The hAAT positive donor cells (brown) are detected surrounding the degraded alginate microsphere (DA) 2 weeks after transplantation; (D) The hAAT positive donor cells are detected surrounding the residual alginate microsphere (RA) 2 weeks after transplantation. (E-F) Validation of the specificity of anti-hAAT antibody: (E) Human liver stained by anti-hAAT antibody as a positive control, whereas (F) Mouse liver stained by anti-hAAT antibody as a negative control (N = 2).

We observed a number of intact alginate microspheres 2 hours after cell transplantation, indicating alginate microsphere were implantable into the liver via injection (Fig 3A and 3B). The hemorrhage due to injection was observed but controllable. All animals under these procedures survived until the end of the study. Interestingly, two weeks after transplantation we observed some degraded alginate microspheres and a number of hAAT positive donor cells were detected surrounding the degraded alginate microspheres (Fig 3C) and residual alginate microspheres (Fig 3D). These results indicated the ADSCs could migrate from degraded alginate microspheres into the liver tissue and survived for at least two weeks. The human liver tissue section was used as a positive control for anti-hAAT antibody (Fig 3E), while mouse liver tissue section was used as a negative control for anti- hAAT antibody (Fig 3F).

### Commitment of hepatogenic differentiation of undifferentiated ADSCs in the liver

**Fig 4:**
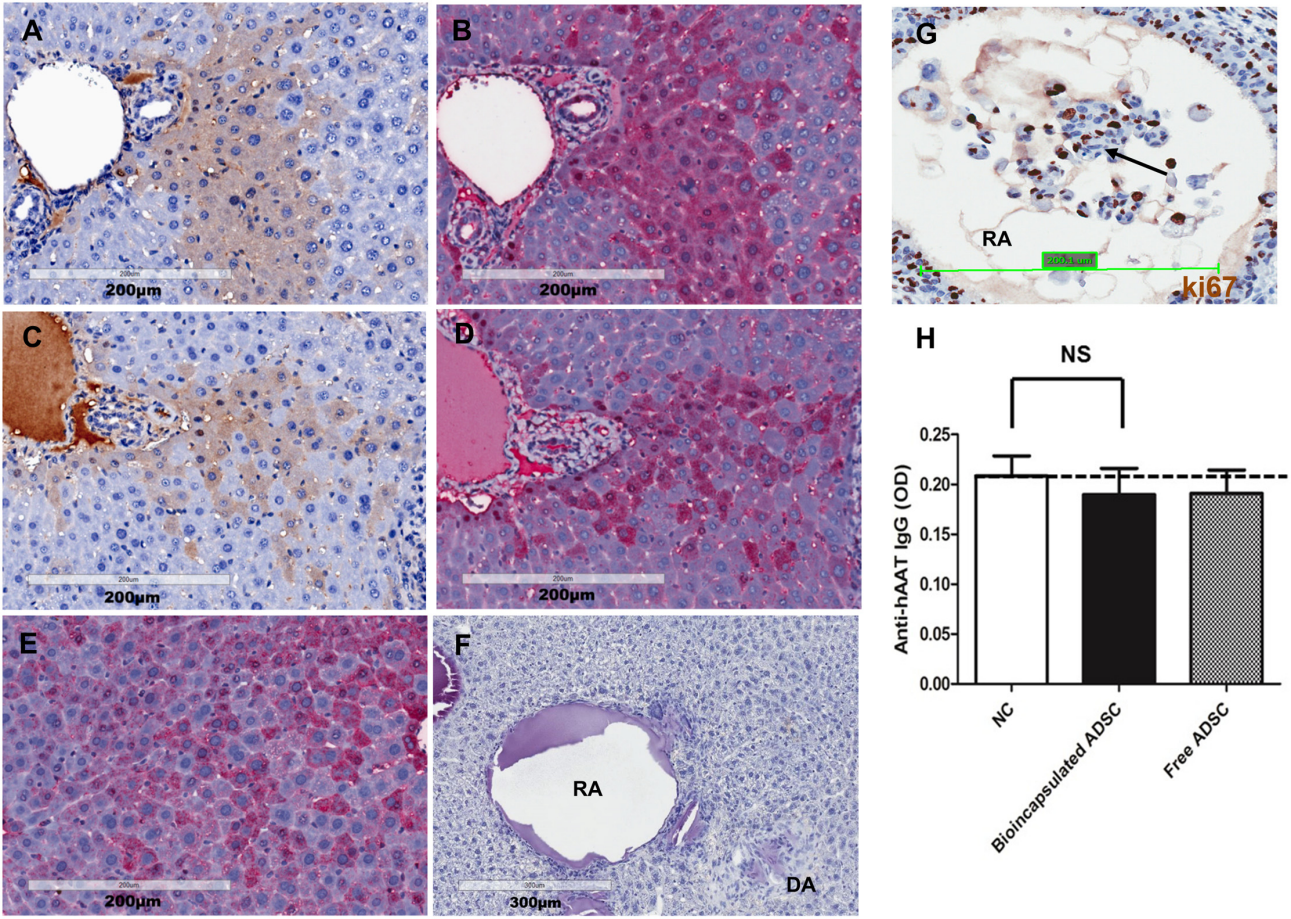
Assessment of the *in vivo* fate of bioencapsulated ADSCs 3 weeks after *in situ* transplantation. (A-D) Hepatogenic differentiation of bioencapsulated ADSCs: mouse liver stained by anti-hAAT antibody (A and C); Mouse liver stained by anti-albumin antibody (B and D), in which B is the consecutive liver section of A, and D is the consecutive liver section of C; (E) Validation of the specificity of anti-albumin antibody: mouse liver (after PH but without cell transplantation) stained by anti-albumin antibody; (F) Evaluation of the interaction between alginate and anti-hAAT antibody: mouse liver containing empty alginate microspheres stained by anti-hAAT antibody; (G) The proliferative activity of ADSCs within the residual alginate microspheres was detected by anti-ki67 antibody. The arrows indicated ki67 positive cells; (H) Evaluation of generation of anti-human AAT antibody by ELISA. Serum samples collected from normal C57BL/6 mice without any treatment were used as a negative control (NC). The dotted line indicated the lower limit of quantification (LLOQ) (N = 4).

Subsequently, in study two we evaluated whether liver microenvironment after PH could induce bioencapsulated, undifferentiated ADSCs toward hepatogenic differentiation and evaluate the possible distribution of survived donor cells in major tissues organs. At the end of this study, we observed a high degree of co-localization between hAAT and mouse albumin in the recipient liver, indicating ADSCs underwent hepatogenic differentiation toward cells express albumin (Fig 4A–4D). The mouse liver tissue section (after PH but without any treatment) was used as a positive control for anti-albumin antibody. Each endogenous hepatocyte expressed albumin at different level (Fig 4E). The interaction between anti- hAAT antibody and alginate is not detectable by hAAT immunostaining (Fig 4F). Further, we observed the cell proliferation within the residual alginate microspheres, as evidenced by Ki 67 positive immunostaining (Fig 4G). In addition, to rule out the possibility that hAAT could induce the generation of anti-hAAT antibody in recipient mice, anti-hAAT antibody ELISA was performed. No anti-hAAT antibody was detected, consistent with our previous observation [12] (Fig 4H).

### Analysis of bio-distribution of transplanted transgenic ADSCs in recipient mice

**Fig 5:**
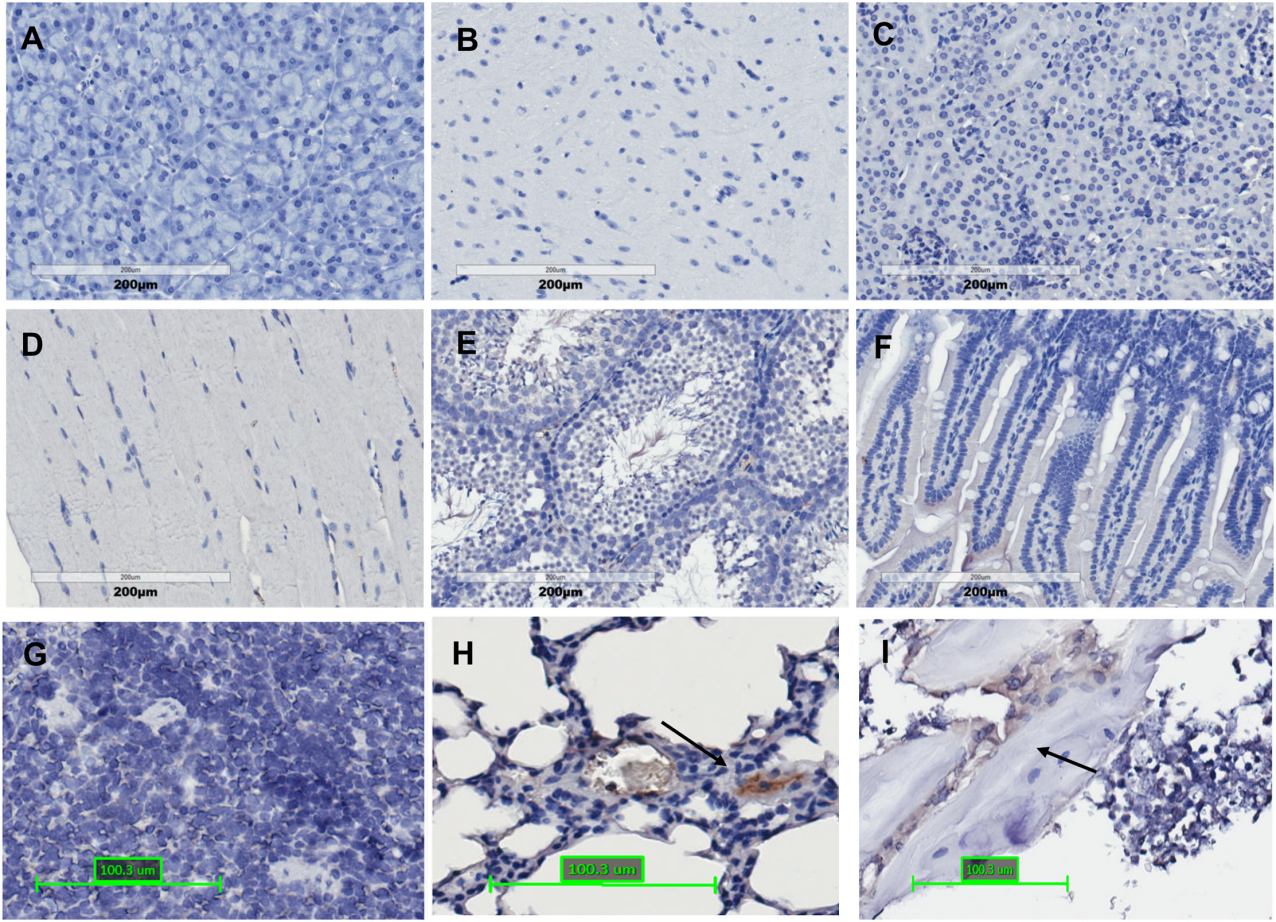
The distribution of bioencapsulated ADSCs in different organs 3 weeks after in situ transplantation. (A) Pancreas; (B) Brain; (C) Kidney; (D) Muscle; (E) Testes; (F) Intestine; (G) Spleen; (H) Lung; and (I) Bone Marrow. The arrows indicated hAAT positive cells (N = 4).

**Fig 6:**
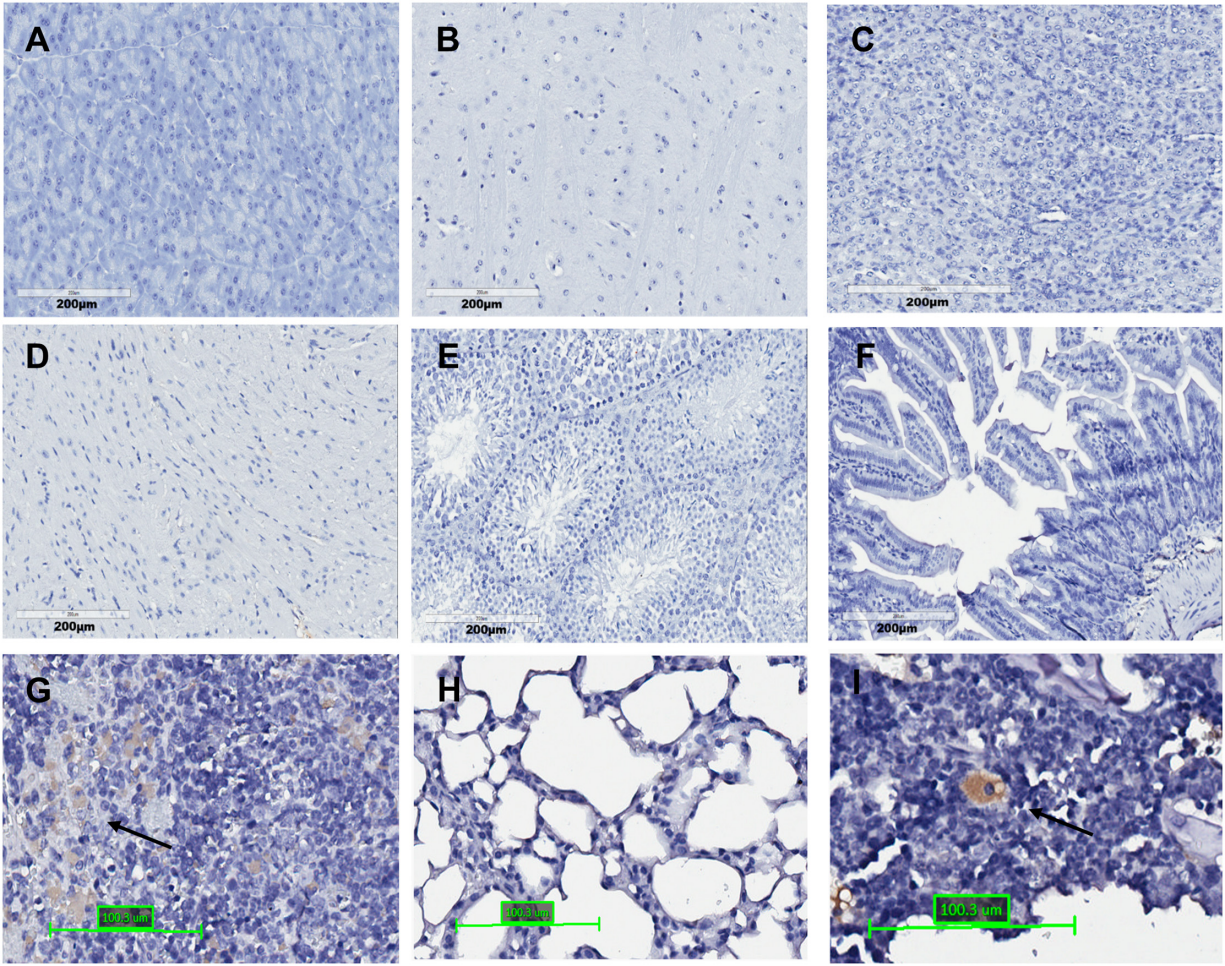
The distribution of free ADSCs in different organs 3 weeks after intrasplenic. injection. (A) Pancreas; (B) Brain; (C) Kidney; (D) Muscle; (E) Testes; (F) Intestine; (G) Spleen; (H) Lung; and (I) Bone Marrow. The arrows indicated hAAT positive cells (N = 4).

Because we have observed that ADSCs could migrate from degraded alginate microspheres in the liver, it is of interest to determine if those surviving ADSCs could migrate to other organs. The biodistribution of transplanted transgenic ADSCs were thus evaluated in major tissue organs including pancreas, brain, kidney, muscle, testes, intestine, spleen, lung, and bone marrow. We found donor cells (hAAT positive cells) are detectable in bone marrow and the lungs if bioencapsulated ADSCs are given via hepatic injection (Fig 5), whereas donor cells are detectable in the bone marrow and the spleen if free ADSCs are given via intrasplenic injection (Fig 6).

## Discussion

Adipose tissue derived stem cells (ADSCs) transplantation has recently gained widespread enthusiasm particularly in the perspective to use them as potential alternative cell sources for hepatocytes in treating liver disease, mainly due to their hepatogenic capability *in vitro* and *in vivo*. To deliver ADSCs into the liver with clinical relevance, some issues needed to be carefully addressed, including providing a therapeutic mass, satisfactory engraftment, and subsequent proliferation of transplanted cells (repopulation). In this study, we test the feasibility of *in situ* ADSCs transplantation by injecting bioencapsulated ADSCs into the liver tissues in a hepatectomized mice model. We showed ADSCs loaded alginate microspheres were implantable into the liver tissue via injection. Both degraded and residual alginate microspheres were observed up to 3 weeks. Further, we demonstrated the viable ADSCs transdifferentiated toward cells expressed albumin in the liver and ADSCs migrated to other organs including the bone marrow and the lungs.

Several different natural polymers have been evaluated as possible scaffolds/carriers for hepatocytes and mesenchymal stem cells (MSCs) *in vitro* and *in vivo*, including collagen, alginate, chitosan, and hyaluronic acid. For example, Y. Zhao et al. created hepatocyte/collagen hydrogel constructs and demonstrated formation of thick and vascularized hepatic tissues *in vivo* after implantation in subcutaneous spaces in rat model [21]. K. Li et al. showed chitosan/gelatin composite can be used for *in vitro* hepatocyte culture [22]. Compared to other natural polymers, we selected alginate as our cell carrier because the biocompatibility of alginate to ADSCs is well-documented. One of the major limitation of cells proliferated with matrix-based transplantation is the high initial cell loss and dysfunction [23]. M. Dvir-Ginzberg et al. showed alginate can be used as scaffold for hepatocytes in liver tissue engineering *in vitro* [24]. R. Yao et al. demonstrated *in vitro* adipogenic differentiation when hADSCs were embedded in alginate and alginate/gelatin microspheres [25]. Moreover, N. Lin et al. demonstrated *in vitro* differentiation of bone marrow-derived MSCs into hepatocyte-like cells in an alginate scaffold [26]. These *in vitro* studies implied differentiation capability of mesenchymal stem cells (MSCs) would not be affected by alginate bioencapsulation. Furthermore, alginate is relatively cheaper compared with collagen, and fabrication of alginate microspheres can be easily scaled up.

In addition to natural polymers, decellularized liver matrix (DLM) in which the endogenous cellular components were removed and the extracellular matrix proteins and vasculature were preservative, has also been evaluated as carrier/scaffold for cells of therapeutic potential. P. Zhou et al. demonstrated DLM provided environment for long-term survival and maintenance of the hepatocyte phenotype after implantation at peritoneal site [27]. R. Ji et al. showed *in vitro* hepatic differentiation of MSCs on DLM and *in vivo* cell engraftment into the host liver when differentiated MSCs were harvested from DML and delivered to animals via tail vein infusion [28]. At this end, the interaction between DLM and ADSCs are still largely unknown, and we think these tissue-engineered constructs would need to be further evaluated in the actual liver tissue in addition to the peritoneal site. A comprehensive discussion of polymers which have been used for liver tissue engineering was well summarized by E. Jain et al [29]. But importantly, to our best knowledge, none of the natural polymers discussed above have been implanted directly into the liver tissue and evaluated for their performance in the liver tissue. We are the first to evaluate *in vivo* fate of ADSCs loaded alginate microspheres in the liver tissue via *in situ* transplantation and investigate the interplay among donor cells (ADSCs), biomaterial (alginate microspheres) and local microenvironment in a hepatectomized mouse model. We demonstrated undifferentiated, bioencapsulated ADSCs can be induced by local microenvironment to transdifferentiate toward cells expressed albumin.

It is widely accepted that the physical properties of the microspheres play an important role in regulating cell behavior and biological response following transplantation. Study showed that alginate microspheres eroded completed in saline *in vitro* in less than 3 weeks regardless of synthesis conditions. But the stability of alginate microspheres was increased by addition of calcium into the culture medium. For example, Simpson, N. E. et al. demonstrated increasing the concentration of the CaCl_2_ solution used at the time of gelation enhanced the strength of the alginate gel network [30]. In the *in vivo* condition, alginate polymers exhibit very slow degradation via hydrolytic, oxidative, or enzymatic mechanism in the absence of chronic inflammation or other abnormal stimuli [31], for example, *in vivo* studies have shown that alginate microspheres are stable for several weeks in different tissues [14, 31]. These results might be explained by the fact that alginate material absorb calcium ions via ion exchange with the local microenvironment in the presence of physiological levels of calcium [31].

In our study both residual and degraded alginate microspheres were found in the liver at the end of the experiments. Although the degradation of alginate microspheres was observed in the liver, the degradation processes were uncontrollable at this end. Several factors might contribute to such degradation. First, the composition of alginate microspheres: we used low viscosity, high mannuronic acid content (LVM) alginate to prepare microspheres. The M (mannuronic acid) chain does not actively participating in cross-linking, so the microspheres prepared by an LVM alginate would be less stable than that prepared by a low viscosity, high guluronic acid content (LVG) alginate. Secondly, the target organ: the liver is a highly perfused organ (i.e. the liver blood flow rate is ~ 1.8 ml/min for mice and ~1L/min for human), and thus after injection, the shear stress might accelerate the degradation of LVM alginate microspheres within the liver. Previous study reported about a twofold increase in total flow immediately after PH in pig model [32]. In our study such increased total flow in the liver after PH would further accelerate the degradation of alginate microspheres *in vivo*.

Liver directed cell transplantation is challenging in animal models because endogenous hepatocytes are capable of proliferating and thus compete with transplanted cells during liver regeneration processes [12]. It has been suggested that transplanted hepatocytes need to display a proliferative advantage over resident hepatocytes to be able to repopulate a recipient liver [33]. Thus, to create a less competitive environment for donor cells, monocrotaline (MCT) with reported toxicity in liver sinusoidal endothelial cells, is commonly used to inhibit endogenous hepatocytes proliferation [34]. Remarkably, we do not use MCT in this study, but still observed a reasonably high number of donor cells surviving in the liver (Fig 3C and 3D). In addition, we also observed Ki67 positive cells inside and outside the residual alginate microspheres (Fig 4G). The (Ki67+) cells inside the microspheres indicated that donor cells were proliferating, whereas the Ki67 positive cells outside the microspheres might indicate proliferating donor cells migrated out from the microspheres and proliferating endogenous hepatocytes stimulated by hepatectomy. Because MCT treatment is prohibited from human use, this study approach without MCT might be a more practicable way for clinical application.

When analyzing albumin expression in liver tissue section, we observed enhanced albumin expression in AAT positive donor cells. This finding is consistent with our previous publication but the underlying mechanism led to this observation is still unclear [12]. In our study, the *hAAT* gene in transgenic M-hAAT mice is driven by CMV enhancer/beta-actin (CB) consecutive promoter, meaning AAT protein will be expressed in all types of cells. It is likely that such overexpressed hAAT up-regulated endogenous albumin gene expression when ADSCs underwent hepatogenic differentiation; however, this hypothesis would need to be tested.

Our results in analyzing donor cell distribution showed that ADSCs homed to bone marrow in both delivery routes (Figs 5I and 6I). Migration and homing to the tissues is influenced by multiple factors including age and passage number of the cells, culture condition, the delivery methods, and others [35]. Although the mechanisms by which ADSCs are recruited to tissues and cross the endothelial cell layer are not yet fully understood, it is possible that chemokines and their receptors, such as CXCR4, are involved.

Some limitations of these studies need further investigation. First, although we showed undifferentiated ADSCs differentiated to cells expressed albumin, whether those cells are fully functional remain unclear. It would be very challenging to determine whether a cell is a true hepatocyte *in vivo* and the systematic assessment of the complete phenotype of hepatocyte-like cells is almost unrealistic. But it is commonly accepted that the demonstration of at least one metabolic function, one synthetic function, and one storage function provided sufficient evidence to define a differentiated cell as a hepatocyte-like cell [36]. Alternatively, the animal model which showed transplanted cells completely replaced the deficient native hepatocytes might provide sufficient evidence of a complete functionality [37].

Additionally, long term studies would be recommended to evaluate the degradation of alginate microspheres in the liver, the host response to those residual microspheres, and the possibility of malignant transformation of mesenchymal stem cells. At this time we do not intend to conclude this method is better than any other ADSCs delivery method because the *in vivo* functionality of ADSCs-derived cells needed to be further investigated. In addition, the outcome of cell transplantation studies varies between different animal liver disease models (i.e. surgical vs. chemical-induced liver damage). The covariates in cell transplantation studies need to be carefully controlled, including different passages numbers of ADSCs used and different end-point biomarkers when different routes are compared. But we demonstrate experimental results of a novel strategy to locally deliver undifferentiated ADSCs into the liver, and use the liver regeneration microenvironment to induce undifferentiated ADSCs toward hepatogenic differentiation. One major advantage of this method is that a similar strategy can be easily scaled up to larger animals, such as rats. Further optimizing cell loading number within the alginate microspheres would maximize the amount of cells which can be locally delivered into the liver tissue in attempt to reach a therapeutic mass, and thereby could improve the use of cell therapy for liver disease.

## Conclusion

In this study we evaluated *in situ* ADSCs transplantation via hepatic injection and investigated the interplay among donor cells (ADSCs), biomaterial (alginate microspheres), and local microenvironment in a hepatectomized mouse model up to 3 weeks. Our study provided insight into the behaviors of encapsulated ADSCs at the site of action at *in vivo* condition. We demonstrated undifferentiated, bioencapsulated ADSCs can be locally delivered into the liver and demonstrated donor cells proliferated within the alginate microspheres after transplantation. More importantly, ADCSs can be further induced by microenvironment to transdifferentiate toward cells expressing albumin. These findings support further research into the use of *in situ* transplantation of ADSCs and pave the way of using cell-based therapy in treating liver diseases.
